# SPOP and CUL3 Modulate the Sonic Hedgehog Signal Response Through Controlled Degradation of GLI Family Transcription Factors

**DOI:** 10.3389/fcell.2021.710295

**Published:** 2021-07-30

**Authors:** Patricia A. Umberger, Stacey K. Ogden

**Affiliations:** ^1^Department of Cell and Molecular Biology, St. Jude Children’s Research Hospital, Memphis, TN, United States; ^2^Integrated Biomedical Sciences Program, University of Tennessee Health Science Center, Memphis, TN, United States

**Keywords:** Cullin 3, GLI2, GLI3, Hedgehog signaling, developmental signaling, transcription regulation

## Abstract

The speckle-type POZ protein (SPOP) functions as a guardian of genome integrity and controls transcriptional regulation by functioning as a substrate adaptor for CUL3/RING-type E3 ubiquitin ligase complexes. SPOP-containing CUL3 complexes target a myriad of DNA-binding proteins involved in DNA repair and gene expression, and as such, are essential modulators of cellular homeostasis. GLI transcription factors are effectors of the Hedgehog (HH) pathway, a key driver of tissue morphogenesis and post-developmental homeostasis that is commonly corrupted in cancer. CUL3-SPOP activity regulates amplitude and duration of HH transcriptional responses by controlling stability of GLI family members. SPOP and GLI co-enrich in phase separated nuclear droplets that are thought to serve as hot spots for CUL3-mediated GLI ubiquitination and degradation. A similar framework exists in *Drosophila*, in which the Hedgehog-induced MATH (meprin and traf homology) and BTB (bric à brac, tramtrack, broad complex) domain containing protein (HIB) targets the GLI ortholog Cubitus interruptus (Ci) for Cul3-directed proteolysis. Despite this functional conservation, the molecular mechanisms by which HIB and SPOP contribute to *Drosophila* and vertebrate HH signaling differ. In this mini-review we highlight similarities between the two systems and discuss evolutionary divergence in GLI/Ci targeting that informs our understanding of how the GLI transcriptional code is controlled by SPOP and CUL3 in health and disease.

## Introduction

Targeted proteolysis maintains cellular homeostasis by providing regulatory control for signal transduction, transcription, metabolism and cell division (Glickman and Ciechanover, 2002; Nandi et al., 2006; Teixeira and Reed, 2013). During embryonic development, the HH pathway functions as a regulator of cell fate determination and signals post-developmentally to maintain tissue homeostasis. HH signaling is controlled in part through proteolysis of downstream transcriptional effectors, Ci in *Drosophila* and GLI proteins in vertebrates. Degradation is promoted by Cullin-RING Ligase (CRL) complexes, composed of a cullin scaffold protein, RING subunit, and ubiquitin-bound E2 enzyme (Pintard et al., 2004). GLIs are targeted through the ubiquitin-proteasome pathway by both CUL1 and CUL3-CRL complexes. Whereas CRL1 facilitates partial proteolytic processing of GLI/Ci in the absence of HH signal, CRL3 triggers complete GLI/Ci degradation in ligand-stimulated cells (Jiang, 2006; Smelkinson and Kalderon, 2006; Wang et al., 2010). Herein we discuss proteolysis of GLI proteins by the ubiquitin-proteasome pathway, focusing on CUL3-mediated degradation via the substrate adaptor SPOP. We examine functional divergence controlling GLI transcription factor degradation between *Drosophila* and vertebrate systems and comment on important open questions regarding the molecular mechanisms controlling GLI-SPOP association.

## Ubiquitin-Proteasome Degradation Pathway

Protein entry into the ubiquitin-proteasome pathway is directed by covalent linkage of ubiquitin to lysine residues of protein targets following stepwise ubiquitin transfer along an E1-E2-E3 enzyme cascade (Hochstrasser, 1996). A limited number of E1 ubiquitin activating enzymes service dozens of E2 ubiquitin carrier enzymes and hundreds of E3 ubiquitin ligases (Semple et al., 2003). The largest subclass of E3 ligases are the CRLs. Eight different cullin scaffolding proteins are expressed in human cells (CUL1-3, 4A/B, 5, 7 and 9), each one interacting with specific RING and E2 subunits to form distinct modular ubiquitin ligase complexes (Petroski and Deshaies, 2005). CRL binding to target proteins is directed through recruitment by substate adaptors (Petroski and Deshaies, 2005).

Both CRL1 and CRL3 complexes are crucial modulators of developmental signaling and contribute to HH pathway regulation. CRL1 complexes target proteins that control cell cycle progression, proliferation, and transcription (Teixeira and Reed, 2013). Their substrate specificity is conferred by associated Skp1 and F-box proteins, which recognize phospho-degrons in substrates such as GLI/Ci (Skaar et al., 2013). CRL3 complexes are more widely used than CRL1 complexes, and degrade proteins involved in myogenesis, neurogenesis, chondrogenesis, osteogenesis, and adipogenesis (Dubiel et al., 2018). CRL3 complexes use BTB superfamily proteins as substrate adaptors (Pintard et al., 2004). The MATH-BTB protein SPOP, which recruits GLIs to CRL3, binds substrates in a multivalent manner to enhance their recruitment into discrete ubiquitination and degradation loci (Zhuang et al., 2009; Marzahn et al., 2016).

### SPOP/HIB Is a Multivalent Substrate Adaptor

**FIGURE 1:**
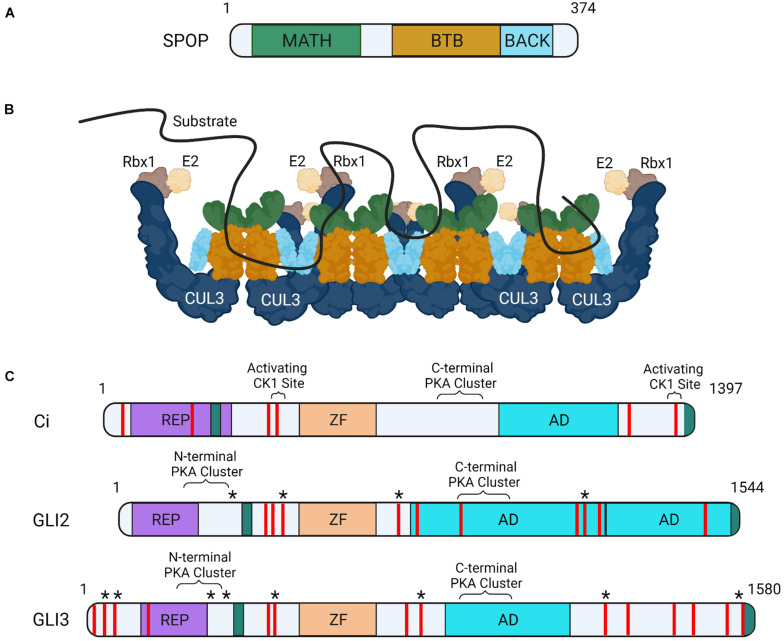
Schematic of SPOP, CRL3-SPOP complex oligomer and substrates Ci and GLI. **(A)** MATH, BTB, and BACK functional domains are indicated on a schematic of mouse SPOP. **(B)** A schematic of CRL3-SPOP complex oligomer with substrate bound through multivalent interactions via multiple linear SBC motifs is shown. Each SPOP substrate adaptor protein (colors correspond to SPOP domains in **A**) is drawn in association with a CUL3 scaffolding, corresponding Rbx1 (RING protein, tan) and E2 enzyme (cream). **(C)** Functional domains of *Drosophila* Ci, mouse GLI2 and human GLI3 proteins are shown. Regions of post-translational modifications influencing transcriptional activity are indicated (known PKA and CK1 sites in brackets, predicted “activating” CK1 sites are indicated with asterisks). SPOP recognition motifs are shown as red lines. SUFU interacting regions (teal), repressor domain (REP, purple), zinc finger domain (ZF, orange), and transcriptional activation domain (AD, blue) are also shown.

SPOP contains three functional domains essential for substrate degradation. These include an amino-terminal MATH domain, a BTB domain and a carboxyl-terminal BACK domain (Figure 1A). The MATH domain binds targets through SPOP binding consensus (SBC) motifs (non-polar-polar-Ser-Ser/Thr-Ser/Thr) (Zhuang et al., 2009). Disordered and elongated SBC motifs within substrates interact with residues of the shallow groove formed by SPOP’s MATH domain (Figure 1B; Zhuang et al., 2009). SPOP substrates contain numerous SBCs, which provide multivalent binding capability required for ubiquitination and degradation through CRL3-SPOP. SPOP’s BTB domain facilitates homodimerization and CUL3 binding and its BTB and C-terminal Kelch (BACK) domain drives oligomerization (Figure 1B; Errington et al., 2012). CRL3 complexes containing SPOP oligomerization mutants have diminished ubiquitination capabilities, supporting that multivalent associations are essential for substrate degradation (Zhuang et al., 2009; Marzahn et al., 2016; Bouchard et al., 2018).

Liquid-liquid phase separation (LLPS), or the de-mixing of proteins in solution, drives formation of large protein assemblies called membraneless organelles. These assemblies are thought to compartmentalize distinct biological processes within a cell for efficient interaction between assembly partners (Banani et al., 2017; Shin and Brangwynne, 2017; Boeynaems et al., 2018). A protein’s ability to undergo LLPS is driven by multivalent interactions. SPOP can undergo LLPS through homotypic interactions, as exemplified by its enrichment in nuclear speckles. SPOP can also phase separate with binding partners including GLI and DAXX (Kwon et al., 2006; Marzahn et al., 2016). Whereas individual SBCs have weak SPOP binding affinities, the presence of multiple SBC motifs within substrates enhance SPOP association by increasing avidity (Figure 1C; Pierce et al., 2016). This promotes higher-order incorporation of SPOP with substrates into large assemblies that condense to drive LLPS for CRL3-mediated ubiquitination (Errington et al., 2012; Bouchard et al., 2018). Substrates that must phase separate with SPOP for efficient ubiquitination include the GLI proteins. SPOP mutants that prevent higher order oligomerization and LLPS fail to efficiently ubiquitinate GLIs, which triggers HH gain-of-function phenotypes *in vivo* (Marzahn et al., 2016).

## The Hedgehog Signaling Pathway

**FIGURE 2:**
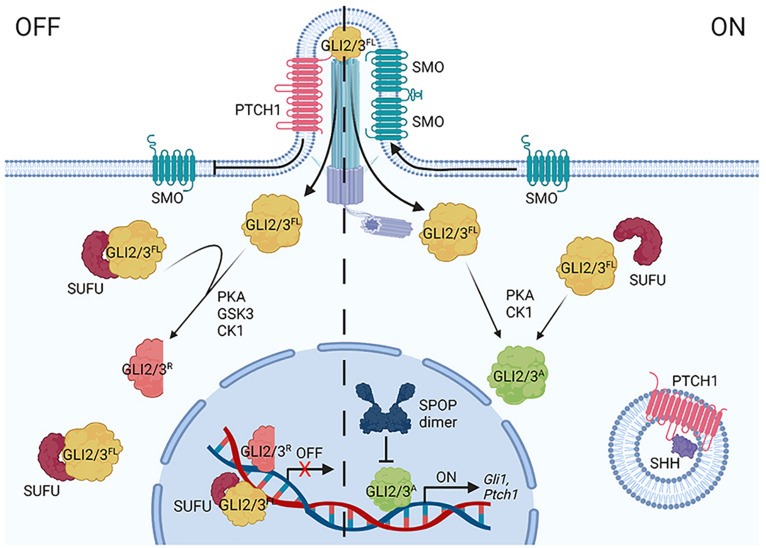
A simplified diagram of the vertebrate HH signaling pathway. In the off state (left), Patched1 (PTCH1) represses Smoothened (SMO) by inhibiting its entry into the primary cilium (PC). GLI2/3 effectors are targeted for partial processing into repressor forms (GLI2/3^*R*^) following sequential phosphorylation via PKA, GSK3(β), and CK1. SUFU binds GLI2/3^*FL*^ in the cytoplasm and nucleus. SHH binding to PTCH1 (right) triggers its endocytosis, enabling SMO to oligomerize and enrich in the PC. GLI2/3 proteins are activated (GLI2/3^*A*^) through alternate phosphorylation events (PKA, CK1), causing dissociation from SUFU and entry into the nucleus to induce target genes (including *Gli1* and *Ptch1*). CRL3-SPOP tunes the GLI response by targeting nuclear GLI2/3^*A*^ for degradation.

HH pathway regulation occurs through a series of inhibitory protein interactions that are reversed by ligand binding (Figure 2). In the absence of HH, its receptor Patched (PTCH) inhibits activity of the signal transducing protein Smoothened (SMO) (Arensdorf et al., 2016). In this off state, GLI2, GLI3 and Ci transcriptional effectors are targeted for partial proteolysis to remove their transcriptional activator domains (Chen et al., 2009; Hui and Angers, 2011). Full-length GLI2/3/Ci (GLI2/3^*FL*^/Ci^*FL*^) proteins that are not immediately truncated bind the tumor suppressor SUFU, which prevents transcription activation in the absence of HH through multiple mechanisms. These include promoting full-length to repressor conversion and preventing nuclear shuttling of GLI^*FL*^/Ci^*FL*^ proteins (Barnfield et al., 2005; Chen et al., 2009; Tukachinsky et al., 2010; Shi et al., 2014a). SUFU has also been reported to chaperone GLI^*FL*^/Ci^*FL*^ into the nucleus and to recruit histone deacetylase activity to nuclear GLI2/3 through binding the mSin3 co-repressor complex member SAP18 (Cheng and Bishop, 2002; Paces-Fessy et al., 2004; Sisson et al., 2006; Zhang et al., 2017).

HH ligand binding to PTCH activates the pathway by inducing PTCH internalization and degradation, allowing for enrichment of SMO dimers/oligomers on the plasma membrane in *Drosophila* and in the primary cilium in vertebrates, where it signals for GLI2/3 stabilization and activation (Figure 2; Bangs and Anderson, 2017; Kong et al., 2019). Full-length activated GLI2/3/Ci (GLI2/3^*A*^/Ci^*A*^) does not efficiently associate with SUFU, and as such, readily enters the nucleus to activate target genes after exiting the primary cilium (Chen et al., 2009; Humke et al., 2010). Whereas *Drosophila* signaling culminates in activation of a single GLI family member, Ci, vertebrates have three HH transcriptional effectors, GLI1-3 (Croker et al., 2006; Briscoe and Therond, 2013). As introduced above, stability and activity of GLI2/3 are directly controlled by pathway induction. The gene encoding GLI1 is a transcriptional target of GLI2/3^*A*^, which is induced in a feed-forward loop to amplify the transcriptional response (Hui and Angers, 2011; Briscoe and Therond, 2013). Unlike GLI2/3^*A*^, GLI1 is not a degradation substrate of SPOP-CUL3 complexes (Zhang et al., 2009), so its regulation will not be further discussed in this review.

### Proteolytic Regulation of GLI/Ci

As introduced above, GLI2/3 and Ci undergo complex degradation cycles that vary depending upon whether a cell is exposed to ligand. In the absence of ligand, GLI2/3 and Ci are targeted for partial proteolysis by the 26S proteasome, converting them into truncated transcriptional repressors (Figure 2, GLI2/3^*R*^/Ci^*R*^) (Aza-Blanc et al., 1997; Jiang, 2006; Wang and Li, 2006). GLI2/3 and Ci proteins are marked for proteolysis through a phosphorylation cascade initiated by cyclic AMP-dependent protein kinase (PKA), which leads to subsequent phosphorylation by glycogen synthase kinase 3 [GSK3(β)] and casein kinase 1 (CK1) (Figure 1C; Price and Kalderon, 2002; Hui and Angers, 2011). Multi-site phosphorylation promotes GLI2/3 and Ci recruitment to CRL1 by the F-box substrate adapter β-TrCP/Slimb (Jiang, 2006; Smelkinson and Kalderon, 2006). Pathway activation shifts GLI2/3 and Ci phosphorylation, attenuating repressor conversion and promoting GLI2/3^*A*^/Ci^*A*^ (Figures 1C, 2; Ohlmeyer and Kalderon, 1998; Hui and Angers, 2011; Niewiadomski et al., 2014).

Once in the nucleus, GLI2/3^*A*^/Ci^*A*^ are candidates for proteolysis by CRL3-SPOP/HIB complexes that limit the GLI2/3^*A*^/Ci^*A*^ transcriptional response (Kent et al., 2006; Zhang et al., 2006). The role for SPOP in regulating stability of GLI family members was first recognized in *Drosophila* following the discovery that the gene encoding the SPOP homolog HIB is a Ci transcriptional target. This led to a model in which HIB powered a negative feedback loop to degrade Ci in cells receiving high-level HH stimulation (Kent et al., 2006; Zhang et al., 2006).

Ci contains at least six SBCs and GLI2/3 proteins contain upwards of ten (Figure 1C; Zhang et al., 2009). SPOP/HIB can bind SBCs in amino and carboxyl-terminal regions of GLI3 and Ci, and the carboxyl-terminal region of GLI2 (Zhang et al., 2009; Wang et al., 2010). Despite the ability of amino-terminal SBCs to associate with SPOP in the context of the full-length proteins, *in vitro* over-expression studies of GLI2/3^*R*^ mimics suggest GLI^*R*^/Ci^*R*^ are not efficiently degraded by CRL3-SPOP (Wang et al., 2010). This differential degradation efficiency of GLI2/3^*FL*^/Ci^*FL*^ versus GLI^*R*^/Ci^*R*^ may result from affinity differences of amino- and carboxyl-terminal SBCs. *In vitro* Ci binding assays revealed that carboxyl-terminal SBCs recruited HIB more efficiently than amino-terminal SBCs (Zhang et al., 2009). However, amino-terminal SBC-HIB binding efficiency increased with forced Ci dimerization, suggesting amino-terminal sites may cooperate in *trans*. SBCs in the carboxyl domain of Ci functioned cooperatively in *cis*, allowing for high affinity combinatorial binding to HIB independent of Ci dimerization (Zhang et al., 2009).

Another mechanism by which SPOP-GLI2/3 and HIB-Ci binding may be affected is through phosphorylation of serine/threonine residues within SBC motifs. Some GLI2/3 and Ci SBCs are phosphorylated by the serine/threonine kinase CK1 following pathway activation (Shi et al., 2014b). Although activating CK1 sites in GLI2/3 have not been mapped, phosphorylation by CK1 is reported to attenuate SPOP/HIB binding to slow GLI2/3^*A*^/Ci^*A*^ degradation. Notably, whereas all Ci SBCs are capable of being phosphorylated by CK1, select SBC clusters are phosphorylated more efficiently (Figure 1C). This suggests graded CK1 phosphorylation may provide a mechanism to tune SPOP/HIB binding proportional to HH exposure (Shi et al., 2014b). SBC phosphorylation in SPOP substrates MacroH2A, Puckered, and Pdx1 prevents SPOP binding, suggesting phosphorylation provides a conserved regulatory mechanism for CRL3-SPOP engagement (Zhuang et al., 2009; Ostertag et al., 2019).

PKA is another serine/threonine kinase with dual roles in GLI2/3 control. In response to SHH, GLI2/3 proteins are phosphorylated by PKA at a series of amino-terminal sites that promote GLI2/3^*A*^ (Figure 1C; Niewiadomski et al., 2014). Like GLI, Ci has multiple PKA clusters in its amino-terminus, suggesting ligand-stimulated phosphorylation plays a conserved role in Ci^*A*^ generation (Niewiadomski et al., 2014). The activating PKA sites in GLI/Ci are located adjacent to amino-terminal SBCs, but whether they directly impact SPOP/HIB binding is not yet clear. Given the ability of CK1 phosphorylation to directly alter affinity of SBCs for SPOP, it is possible that PKA phosphorylation could have similar effects (Shi et al., 2014b). Alternatively, PKA phosphorylation may alter SPOP binding indirectly by inducing conformational shifts in GLI/Ci that hinder multivalent SPOP/HIB association.

SUFU binds GLI2/3^*FL*^/Ci^*FL*^ proteins in both flies and vertebrates. In addition to buffering GLI2/3/Ci transcriptional activity through this binding, SUFU also buffers against SPOP/HIB. This is because SBCs in GLI2/3/Ci are located adjacent to SUFU recognition sites, which results in SUFU and SPOP/HIB binding GLI2/3/Ci in competitive manners (Dunaeva et al., 2003; Zhang et al., 2009; Seong and Ishii, 2013; Han et al., 2015). A role for SUFU in blunting SPOP-mediated GLI2/3 destabilization was delineated in mouse embryo fibroblasts (MEFs) derived from *Sufu* knockout animals. Reduced levels of both GLI2/3^*A*^ and GLI3^*R*^ observed in the absence of SUFU were rescued by its re-expression (Chen et al., 2009; Wang et al., 2010). So, while both SUFU and SPOP negatively regulate GLI2/3 activity, they do so through distinct mechanisms. Whereas SPOP limits the duration of GLI2/3^*A*^ transcriptional activity by degrading GLI2/3^*A*^ in the nucleus, SUFU controls the amplitude of the GLI transcriptional response through physical association with GLI to block both CRL3-SPOP degradation and transcriptional activity.

## CRL3-HIB Regulation of *Drosophila* HH Signaling

A role for CRL3-HIB in controlling Ci stability was identified through misexpression studies during *Drosophila* eye and wing development. Ectopic expression of HIB reduced Ci^*FL*^ protein in both tissues, and HIB elimination resulted in Ci^*FL*^ accumulation and increased target gene induction (Kent et al., 2006; Zhang et al., 2006). These results led to the model whereby HIB induction limits Ci^*A*^ accumulation and activity in cells receiving high-level HH stimulation. HIB-mediated Ci degradation in the wing was initially proposed to be spatially restricted due to high-level HIB expression occurring only in cells directly adjacent to a HH source (Kent et al., 2006; Zhang et al., 2006). However, over-expression studies revealed HIB limits wing overgrowth caused by excessive Ci transcriptional activity throughout the wing, suggesting its ability to control Ci is not spatially limited (Zhang et al., 2006).

Because HIB is the only MATH-BTB protein in *Drosophila*, and it is induced by high-level HH signaling, it was initially proposed to be a specific modulator of Ci. However, more recent studies demonstrate HIB influences stability of proteins not linked to the HH cascade including the phosphatase Puckered, and the chromosomal proteins CAL1 and Histone H3 variant CENP-A (Liu et al., 2009; Zhang et al., 2009; Bade et al., 2014). Additionally, a recent report revealed CRL3-HIB regulation of Ci is not as straightforward as indicated by early over-expression studies. CRISPR-generated *Drosophila* harboring mutations in the endogenous *ci* allele that disrupt HIB binding develop normally, suggesting HIB is not essential for regulation of Ci expressed at physiological levels (Little et al., 2020). The cause of these disparate results is not yet clear but may indicate that use of over-expression systems can mask cooperating or redundant mechanisms that control Ci activity. This raises the possibility that HIB may not function as a specific feed-back regulator of HH signaling in flies but may instead prevent excessive signaling when Ci accumulates to uncharacteristically high levels.

## CRL3-SPOP Regulation of Vertebrate HH Signaling

Despite the conserved role of CRL3-SPOP/HIB in controlling the amplitude and duration of HH responses through ubiquitination and degradation of GLI2/3^*A*^/Ci^*A*^, divergence between the systems has occurred. Most notably, whereas CRL3-HIB degrades Ci^*A*^ independent of tissue context, CRL3-SPOP regulation of GLI2/3^*A*^ occurs in tissue and context-dependent manners (Cai and Liu, 2016, 2017; Coquenlorge et al., 2019; Yin et al., 2019). In the neural tube (NT), Sonic Hedgehog (SHH) instructs organization of ventral neuronal progenitor domains through balancing activity of GLI^*A*^ and GLI^*R*^ proteins (Persson et al., 2002; Lei et al., 2004). Despite the ability of CRL3-SPOP to directly target GLI2/3^*A*^, the ventral NT is appropriately patterned in *Spop* knockout mice, suggesting SPOP is not an essential regulator of GLI2/3 in this tissue (Cai and Liu, 2017). However, individually targeting *Gli2* or *Gli3* in *Spop*^–/–^ mice revealed SPOP specifically impacts GLI3^*A*^ during NT development. Failure of ventral NT fate induction observed in *Gli2^–/–^* animals was rescued in *Gli2/Spop* double knockout animals, indicating SPOP loss may increase compensatory GLI3^*A*^ activity in the absence of GLI2. Indeed, increases in both GLI3^*FL*^ and GLI3^*R*^ were observed in *Spop^–/–^* tissue (Cai and Liu, 2017). Importantly, comparison of *Gli3^–/–^* and *Gli3/Spop* double knockout embryos revealed similar NT defects. This supports that GLI3 is likely the main target of SPOP in the NT, and that CRL3-SPOP is not a key modulator of GLI2^*A*^ protein levels in this tissue (Cai and Liu, 2017).

*Spop^–/–^* mice reveal that, in bone, SPOP plays a positive regulatory role that promotes GLI3 activity downstream of Indian Hedgehog (IHH). The positive contribution of SPOP was identified through skeletal target gene analysis, which revealed reduced IHH target gene induction and decreased osteoblast and chondrocyte differentiation in *Spop^–/–^* metatarsal and limb tissues (Cai and Liu, 2016). Similar to what was observed in the *Spop^–/–^* NT, effects on GLI2 were modest in the skeleton. Intriguingly, SPOP loss impacted IHH signaling by increasing GLI3^*R*^, rather than by influencing GLI3^*A*^ (Cai and Liu, 2016, 2017). Whether this indicates direct targeting of GLI3^*R*^ by SPOP in skeletal tissue, or if GLI3^*R*^ accumulation was the byproduct of increased full-length GLI3, is not yet clear. CRL3-SPOP may directly target GLI3^*R*^ because amino-terminal constructs that mimic GLI3^*R*^/Ci^*R*^ can be ubiquitinated by CRL3-SPOP/HIB in *in vitro* assays (Zhang et al., 2006; Cai and Liu, 2016; Marzahn et al., 2016). Further investigation is needed to clarify whether GLI3^*R*^/Ci^*R*^ are *bona fide* physiological targets of CRL3-SPOP.

Specific effects on GLI2^*A*^ by CRL3-SPOP were identified in the mesenchymal niche and brain following combined targeting of *Sufu* with *Spop*. Gut mesenchymal development depends upon coordinated activity of SHH, IHH and platelet-derived growth factor signaling between mesenchymal and epithelial layers (Mao et al., 2010; Walton et al., 2012). Activation of downstream signaling by conditional deletion of *Sufu* in gut mesenchyme resulted in enlarged stomachs, shortened intestines, significant mesenchymal tissue expansion, and perinatal lethality. Whereas conditional deletion of *Spop* in the gut mesenchyme did not yield a developmental phenotype, combining *Spop* with *Sufu* deletion exacerbated the *Sufu* knockout phenotype (Coquenlorge et al., 2019; Yung et al., 2019). In *Spop/Sufu^–/–^* animals, elevated GLI2 led to mesenchymal and epithelial tissue overgrowth and intestinal tumorigenesis, which was corrected by reducing *Gli2* gene dosage (Coquenlorge et al., 2019).

In the brain, compound deletion of *Spop* and *Sufu* in the cerebellum led to rapid development of highly aggressive medulloblastoma (Yin et al., 2019). Tumors were significantly affected by gene dosage because reduced tumor burden was observed in *Gli2* heterozygous *Spop;Sufu* double knockout animals. Tumors were further reduced by increasing *Spop* gene dosage. *Sufu*−/−;*Spop* + /−;*Gli2* + /− animals showed normal cerebellar patterning and a significant reduction in tumor incidence (Yin et al., 2019). Combined with the work discussed above, these genetic studies suggest SHH pathway activity can be controlled through coordinated targeting of GLI2 by CRL3-SPOP and SUFU to scale transcriptional responses downstream of ligand.

## Concluding Remarks

Complex regulatory events controlling proteolysis of GLI family transcription factors are crucial determinants of HH target gene regulation in the absence and presence of ligand. Experimental evidence indicates CRL3-SPOP complexes are important players in this process but understanding of the molecular mechanisms and context-dependent cues controlling GLI2/3 recruitment to CRL3 by SPOP is in its infancy. As such, continued genetic, biochemical, and biophysical interrogation of CRL3-SPOP regulation of HH pathway activity is needed. A focus area that will yield important information about regulation of GLI family proteins will be dissection of signals driving their LLPS with SPOP. Such studies will reveal how LLPS promotes CRL3-mediated elimination of GLI2/3^*A*^ and may provide novel therapeutic opportunities for targeting aberrant GLI2/3 transcriptional activity in disease. In addition, continued dissection of the coordination between SUFU and SPOP association with GLI2/3/Ci will be essential to clarify the complex regulatory mechanisms controlling GLI2/3/Ci transcriptional output. Such studies may reveal developmental, tissue, and/or temporal-specific cues that direct differential CRL3-SPOP targeting of GLI that may be amenable to therapeutic intervention.

## Author Contributions

PU wrote the manuscript. SO edited the manuscript. Both authors contributed to the article and approved the submitted version.

## Conflict of Interest

The authors declare that the research was conducted in the absence of any commercial or financial relationships that could be construed as a potential conflict of interest.

## Publisher’s Note

All claims expressed in this article are solely those of the authors and do not necessarily represent those of their affiliated organizations, or those of the publisher, the editors and the reviewers. Any product that may be evaluated in this article, or claim that may be made by its manufacturer, is not guaranteed or endorsed by the publisher.
